# Multilingualism and fMRI: Longitudinal Study of Second Language Acquisition

**DOI:** 10.3390/brainsci3020849

**Published:** 2013-05-28

**Authors:** Edna Andrews, Luca Frigau, Clara Voyvodic-Casabo, James Voyvodic, John Wright

**Affiliations:** 1 Linguistics Program, Department of Slavic & Eurasian Studies, Duke University, Durham, NC 27708, USA; E-Mail: cal.wright@gmail.com; 2 Department of Statistical Science, Duke University, Durham, NC 27708, USA; E-Mail: frigau@unica.it; 3 Brain Imaging and Analysis Center (BIAC), Duke University, Durham, NC 27710, USA; E-Mails: cv69@st-andrews.ac.uk (C.V.-C.); jim.voyvodic@duke.edu (J.V.); 4 Department of Radiology, Duke University, Durham, NC 27710, USA

**Keywords:** language, fMRI, multilingualism, second language acquisition, longitudinal studies of language acquisition, proficiency, neurolinguistics, MANCOVA, the Russian language

## Abstract

BOLD fMRI is often used for the study of human language. However, there are still very few attempts to conduct ***longitudinal*** fMRI studies in the study of language acquisition by measuring auditory comprehension and reading. The following paper is the first in a series concerning a unique longitudinal study devoted to the analysis of bi- and multilingual subjects who are: (1) already proficient in at least two languages; or (2) are acquiring Russian as a second/third language. The focus of the current analysis is to present data from the auditory sections of a set of three scans acquired from April, 2011 through April, 2012 on a five-person subject pool who are learning Russian during the study. All subjects were scanned using the same protocol for auditory comprehension on the same General Electric LX 3T Signa scanner in Duke University Hospital. Using a multivariate analysis of covariance (MANCOVA) for statistical analysis, proficiency measurements are shown to correlate significantly with scan results in the Russian conditions over time. The importance of both the left and right hemispheres in language processing is discussed. Special attention is devoted to the importance of contextualizing imaging data with corresponding behavioral and empirical testing data using a multivariate analysis of variance. This is the only study to date that includes: (1) longitudinal fMRI data with subject-based proficiency and behavioral data acquired in the same time frame; and (2) statistical modeling that demonstrates the importance of covariate language proficiency data for understanding imaging results of language acquisition.

## 1. Introduction

The question of how language or languages are represented in the human brain is one of the more challenging problems of contemporary neuroscience and neurolinguistics. The following study is unique in its attempt to follow the acquisition of Russian language in a longitudinal study that combines fMRI data analysis with extensive empirical language proficiency data of the subjects. 

The important unique aspects of this study include: (1) the collection and analysis of *longitudinal* data of multilinguals for listening comprehension and reading; (2) the ecologically-valid design of the experiment; (3) the inclusion of empirical data of the language proficiency of the subjects in five areas (reading, listening comprehension, writing, grammar and speaking) in terms of embedded testing in course work, as well as internationally recognized and comparable scales of measurement for all of the languages used in the study developed by the Council of Europe; and (4) the focus on discourse-level language function.

Some of the issues that inform our research group include a careful redefinition of human language in the context of the neurosciences, new approaches to the study of language acquisition and maintenance in bi- and multilingual subjects using imaging technologies, and the development, implementation and analysis of protocols involving listening comprehension and reading in four languages (Germanic language [English], Slavic language [Russian], Romance language [Spanish], Caucasian language [Georgian]).

### 1.1. Defining Human Language

Our working definition of human language focuses on language as a *dynamic*, ***hierarchical***, ***and learned relatively-autonomous system of meaning-generating paradigmatic and syntagmatic signs that signify and communicate via speech communities and communities of practice to self and others throughout the life cycle***. Such a definition captures important principles of language as a cultural phenomenon, as well as a neurological one. Human language is not manifested *in the one* and does not develop normally outside of the cultural context. The fundamental research goals that inform the construction, conducting and analysis of our fMRI longitudinal study of second language acquisition and maintenance include: (1) a sophisticated view of how language operates in the brain (including phonological, morphological, lexical and discourse levels) based on both theoretical linguistic principles and neurolinguistic research; (2) data-driven experimental models for testing our hypotheses about brain and language (both with and without the use of technologies in our measurements); and (3) focus on bi- and multilingual subjects.

### 1.2. Context of the Longitudinal Study

This introductory analysis includes a focus on five of seven subjects who undertook intensive acquisition of contemporary standard Russian in a 10-month period (August 2011–June 2012) at Duke University (USA) and St. Petersburg State University (Russia). In addition to intensive language study, students were also enrolled in a theoretical linguistics course and a neurolinguistic course devoted to brain and multilingualism during the Fall, 2011 semester. The imaging data for the first three scans for each subject spans a one-year period (from April 2011 to April 2012). We focus our initial analysis on five of the subjects (2 male/3 female, 19–20 years of age) since they had no prior exposure to Russian and began their intensive program of Russian language study at the end of August, 2011, which continued through the summer of 2012. The other two had already begun their non-intensive study of Russian previous to this time, where one was beginning the second year sequence and the other the fourth year sequence. All seven subjects participated in a three-week submersion experience at St. Petersburg State University in Russia (October–November, 2011) with a total of 60 contact hours at the appropriate level. In January, 2012, six of the seven subjects were enrolled in an intensive second year Russian language course at Duke University, while the seventh subject continued study in the fourth year sequence. All seven participated in language and culture study at St. Petersburg State University (Russia) for a second in-country intensive experience with 120 formal training contact hours at the university and extracurricular programming for a period of 52 days from May 7 to June 28, 2012. All subjects were L1 English speakers and educated in schools where English was the language of instruction (excluding study abroad experiences).

There has been a great deal of criticism of fMRI language studies in the past due to the absence of extensive information of the subjects themselves, and their exposure/learning trajectory of the languages in question. We hope to address many of these issues in the present analysis by using covariance statistic methods of analysis that incorporate proficiency testing data on each of the subjects during the scanning study. There are six central issues mentioned in de Bot that are relevant to appropriate presentation of data that are essential for contextualization of the imaging results: (1) intensity of contact; (2) motivation to learn the new language; (3) language aptitude; (4) attitudes by learners toward the L1, L2, L3, *etc.;* (5) other languages learned by subjects prior to the study; and (6) degree of literacy of subjects [1]. 

### 1.3. Fundamentals of the Common European Framework Proficiency Scale and Specifics of LfMRI SLAM

In our study, we recorded testing data from coursework and the Russian Federation Language Proficiency Exam (TRKI). TRKI is part of the Common European Framework CEFR scale, which divides learners into three broad divisions and six levels, noted in [2,3]:

**A: Basic User **
**A1** Breakthrough or beginner; **A2** Waystage or elementary
**B: Independent User **
**B1** Threshold or intermediate; **B2** Vantage or upper intermediate
**C: Proficient User **
**C1** Effective Operational Proficiency or advanced; **C2** Mastery or proficiency

The Russian Ministry of Education Language Exam for Russian as a Foreign Language uses these six levels in five categories—speaking, reading, writing, listening comprehension, grammar/lexicon. Our subjects were proficiency tested in November, 2011, December, 2011, January, 2012 and June, 2012 at the A2 and B1 levels. Scores used in constructing covariance tests include listening comprehension, grammar, reading and writing. All subjects passed an oral proficiency battery in August/September 2012. Scores for all written aspects of the proficiency examination process (listening comprehension, grammar, reading, writing) were included in the multivariate analysis of covariance along with activation results from the set of longitudinal scans.

Formal language instruction constituted 108 hours in the fall semester 2011, 60 h in the spring of 2012 and 120 h in the summer of 2012, yielding 288 contact hours. Each subject spent a total of 73 days in St. Petersburg, Russia during that period. All of the subjects were either second or third year college students, and they were highly motivated to learn Russian as participants in this unique submersion program. While we cannot speak specifically to language aptitude, there are two important aspects that are related to such aptitudes, including: (1) overall academic performance; and (2) their previous language study and testing scores. In this regard, it is interesting to note that all of the subjects have high GPAs in their respective majors. The following chart presents the languages and levels of second and third language proficiency that was found among the subjects at the beginning of the longitudinal study**:**

**L2/L3 proficiency of five subjects:**
French/C1, AP 5, SAT II-800, Italian/B1
German/B2/C1, AP 5, IB HL-6, Arabic/B1
French/A1, Italian/A1
Spanish/A2
Spanish/SAT II, AP 5

## 2. Background and Significance

The fundamental controversies surrounding the study of language and brain in research can be summarized into three primary sets of questions:

(1) What part of human language, if any, is innately given in the organism? While everyone agrees that some degree of learning is necessary, to what extent is language learning “natural”?

(2) What is the degree of autonomy of language structures in the human brain? There are **modularity** and **connectivist** approaches informed by varying degrees of commitment to localization and non-localization hypotheses of cognitive structures.

(3) What is the relevance of critical periods for language acquisition and language learning? Definitions of critical periods necessarily differ for different brain regions and different cognitive functions. Dowling refers to these periods as “periods of more susceptibility” that “can be modified by environment” [4]. For example, it would be inappropriate to treat as comparable critical periods for visual cortical structures and acquisition of a first or second/third language. 

These three important sets of questions serve as a central part of the theoretical orientation behind the construction of the experiments and protocols designed as part of our **fMRI longitudinal study of second language acquisition and multilingualism** (henceforth LfMRI SLAM). (For a more detailed discussion of these issues, see [5].)

### 2.1. Results from Previous Studies

The field of neurolinguistics has been heavily defined by the study of language pathologies. Furthermore, it has been generally uncommon until recently for researchers outside of the medical community to be involved in mainstream neurolinguistics. While we share with many other studies a focus on normative language usage and acquisition, we also have a research design that is theoretically informed and sensitive to important advancements in terms of the field of general linguistics, including cognitive and sociolinguistic paradigms, and that includes robust empirical data on subject language proficiency as a fundamental component of an attempt to provide a valid interpretation of scanning results. Furthermore, we have designed protocols that are *ecologically valid* with a focus on longitudinal data collection. It is our goal that the results of these studies will be a contribution to the study of multilingualism and the brain and be stepping stones to new protocols and methodologies to reach deeper levels of analysis and clarity in the field. (Ecological validity of language-based experiments means utilizing language samples in the protocol that do not distort normal language usage at the discourse level and the level of speech acts. For an example of discourse level analysis and speech acts, see [6].)

Important figures in the field of current neurolinguistics whose research is central to the study of multilingualism and neuroscience include: (1) Ojemann and his school where Cortical Stimulation Mapping (CSM) has played a major role in data collection of neuronal firing during language tasks; (2) Poeppel, Hickok and their teams of colleagues who are working to map theoretical linguistic knowledge and neurobiological and neurophysiological data using a variety of imaging technologies, especially MEG and fMRI; (3) Paradis and his colleagues working on bilingual aphasia and testing of bilingual aphasias; (4) a group of important scholars who have done important work in metaanalysis of fMRI and PET, including Price, Binder and Cabeza; and (5) theoretical linguists and neurolinguists who study bilingualism and multilingualism, including de Bot and Bialystok. Their combined works (see [1,6,7,8,9,10,11,12,13,14,15,16,17,18,19,20,21,22,23,24]) provide important baseline results about properties of speech/motor realizations of language and their cortical representations. The work of Ojemann and his team with multilingual patients serves as a significant starting point for re-imagining and reformulating the older views of language and brain and forging a new path for the study of language and brain where **linguistics **is reinserted into the study of **neurolinguistics. **Poeppel and Hickok 2004 provide an important clarification on the current state of research on language and brain, the weaknesses of the “underspecified” traditional model, commonly referred to as the Broca/Wernicke model, and important principles for taking this research to a new level of sophistication with robust results [11]. While both the lesion-deficit tradition and data from healthy subjects play a major role in the field, it is still generally the case that while many linguists are critical of the imaging studies that are done, they rarely do these studies themselves, and the physicians and researchers who do the imaging studies focus on the imaging data and do not often grapple with interpretations and explanations of the results that are more deeply embedded in and compatible with linguistic theories and paradigms. The series of papers resulting from LfMRI SLAM will attempt to do justice to both sets of questions.

The question of neural organization of language centers and language-related areas in the brains of bilinguals and multilinguals is currently a topic of great interest, represented by the significant number of fMRI, PET, EEG and MEG studies found in the recent literature (including [1,15,25,26,27,28,29,30,31,32,33,34,35,36,37,38,39,40,41,42,43,44,45]). Ojemann’s important data obtained through *cortical stimulation mapping* (*CSM*) during surgeries on epileptics, including bilingual patients, provides significant evidence of variation across individuals for motor speech and comprehension that has been crucial to moving the field forward to a new level of hypotheses and research on language and brain. 

A number of previous studies focus only on monolinguals, or at least involve stimuli from only one language ([29,32,46,47]). In those studies that use bilingual ([38,48,49,50,51]) or multilingual ([37,45,52,53]) subjects, there is little proof that the subjects possessed superior or native proficiency in the languages in which they were supposed to be multilingual. Nor was there assurance that parity in multi-language facility existed throughout the research subjects. What has been missing from most of the studies done heretofore is a more linguistically-sensitive and precise evaluation of participants’ abilities in one or more languages prior to submitting these participants to fMRI procedures. Furthermore, earlier studies have done little to quantify linguistic proficiency. For the purposes of our study, subjects participate in a battery of proficiency-based testing to establish their precise levels of linguistic ability. This study uses the international scales acknowledged by the Council of Europe, CEFR, and various government and academic institutions in the United States ([2,3]). Detailed information on the subjects includes a full background of their language knowledge prior to the beginning of the longitudinal study with tracking of their acquisition throughout the period of the study. For those subjects who provide baseline information as highly competent bi- or multilinguals, we provide detailed information about not only their backgrounds, but we also include important data on the degree of daily usage of the languages under analysis and important proficiency data or other forms of verification of their abilities in multiple modalities. Different types of proficiency (audition, speaking, writing, grammatical and lexical knowledge) will be discussed.

Previous studies that address the linguistic abilities of bilinguals and multilinguals are often stratified according to the role of the subject and the nature of the stimuli. In many cases, subjects read, speak, and listen during the data collection phase ([14,38,51,54,55,56,57,58,59,60,61]). In a similar vein, previous studies (see [32,61,62,63,64,65,66,67,68]) have tried to concentrate on particular phonemes or lexemes, or on specific syntactic structures. Our research explores the neural organization of language in the brain by focusing specifically on one (as opposed to three) linguistic tasks at a time. The first stage of the fMRI study was restricted to audition, but a reading component was incorporated into the protocol within the first year of the longitudinal study.

Price’s excellent review of 100 fMRI studies from 2009 divides the results into six different types of protocols, focusing on: (1) prelexical processing; (2) pseudowords; (3) words; (4) sentence comprehension; (5) semantic/syntactic ambiguity; and (6) word retrieval and articulation ([14], p. 65). Price also provides clarification of anatomical ambiguities across the studies with a diagram that maps regions of interest and includes approximations of Brodmann areas ([14], p. 64). Price notes that she did not include multilingual studies in her meta-analysis, and focuses more on left hemisphere activation foci ([14], p. 65, p. 83). While none of the studies are similar enough to LfMRI SLAM to allow for strict comparison within one of the categories given above, we nonetheless pay special attention to Price’s data on sentence comprehension in the discussion.

### 2.2. Methodological Considerations

#### 2.2.1. Research Questions: Hypotheses and Rationale

The primary goal of this study is to achieve a deeper understanding of the neural organization of language-related areas in multilinguals. In order to achieve this goal, the present study will specifically test, and reformulate, the following questions:

(a) Is there a significant neurological variation in the organization of language-related areas in the brains of multilingual subjects who are equivalent in language facility and age of acquisition (pre-adolescent)? Previous research has suggested that “early” bilingual or multilingual acquisition is represented differently in the brain than “late” second or third language acquisition ([31,37,38,39,40,41,42,43,44,45,46,47,48,49,50,51,52,69]). These studies have produced results that are not consistent and inconclusive. The data collected from longitudinal studies of multilingualism will be an important contribution to solving this controversy and providing a more dynamic and empirically-valid view of neurological representations of language(s) in the human brain.

*The use of terms like “early” and “late” are very problematic in the literature. The range of ages included in each term may be distinct or overlapping. In the current study, we will attempt to implement the corrections noted in de Bot (2008) in discussing our subject pool and their trajectory of second, third and fourth language acquisition* [1]*.*

(b) What types of changes will occur within a single subject during periods of language acquisition and maintenance over a period of one, two or more years?
(c) How can fMRI facilitate an understanding of *how* (not merely *where*) language is acquired and maintained neurologically?
(d) How well do the behavioral and imaging data map onto each other?
(e) Many researchers have argued for a more bilateral model of language and brain (*cf.* [11,12]). What does our evidence contribute to this conversation?
(f) Some research has suggested that there is a difference in language-related areas in the brains of multilinguals depending on age of acquisition ([69,70]). We will address the definition of age as a variable using de Bot ([1,20]) and will compare subjects in our study who have used two or more languages across different age spans and in different contexts to test for support or lack therein for such a claim. We hypothesize that proficiency may be a more important variable than biological age.

#### 2.2.2. Method, Design and Procedures

##### 2.2.2.1. Procedure

This experiment uses the CIGAL [71] software package for auditory and visual stimulation as well as real-time recording of subject responses, cardiac and respiratory physiological oscillations, and eye-tracking behavior.

Subjects listen to 30-s recordings of digitized auditory segments of speech in four languages during the session. The stimuli are presented in the audio task in the following way: Language 1 (30 s), Rest (10 s), Language 2 (30 s). This sequence is repeated four times in each run. For the present analysis, we are considering only the English / Russian auditory data. Although the functional scans themselves take approximately 16–17 min, scanning sessions are scheduled for 60 min to include the necessary paperwork, pre- and post-instructions, setup, the anatomical scans, and extra time to reset the scanner between runs.

Pre-scan briefing includes the following instructions to subjects: (1) remain as still as possible; (2) do not open your eyes during the auditory runs; (3) do not “talk to yourself in your head” (*i.e.*, do not use sub-vocalizations). Directly following the sessions, subjects participate in a debriefing session including a list of questions (*cf.* number 8 in Stimuli and Presentation Parameters).

##### 2.2.2.2. Imaging Parameters

Imaging is performed using a General Electric LX 3T Signa scanner using head restraints to reduce motion. All scans discussed herein were conducted on the same **BIAC3 **scanner at Duke University Hospital. The subject’s head is positioned along the canthomeatal line. For functional scans, a total of 30 slices with a 5 mm thickness are obtained using a TR of 1500 ms, a TE of 35 ms, a 64 × 64 matrix, and an echoplanar pulse sequence. Slices are axial, taken parallel to the plane of the anterior and posterior commissures with the most inferior image level with the top of the pons. High resolution dual echo proton density and T2-weighted anatomical images are acquired in the same slice planes as the functional scans. A high resolution 3D fast spoiled GRASS T1-weighted scan covering the whole brain with isotropic 1mm cubic voxels is acquired to allow structural visualization in any orientation (256 × 256 × 166 voxels). 

##### 2.2.2.3. Stimuli and Presentation Parameters

(1) *Digitized auditory segments*: Four languages (Russian, English, Spanish, and Georgian) played through headphones in 30-s blocks with two alternating languages in each run. (Students wear headphones in the scanner. The sound quality is good enough to be heard over the scanner noise. We ensure that this is the case with testing of the stimuli before scanning and through post-scan interviews with subjects). In the scanning sessions with the reading task included, the auditory segments included three languages (Russian, English, Spanish).
(2) The subjects are told in advance only that they will be hearing samples of different languages. The voices for each language will be different (including male and female voices at an indeterminable age [*i.e.*, no child or elderly voices will be used]) but with native pronunciation.
(3) The speakers recorded in the digital sound files were unknown to the subjects participating in the study. In the longitudinal study, subjects hear the same protocol across scans. However, any habituation effects are unlikely, given the large time frames between sessions. Using the same stimulus files at each visit is important for the purposes of this study so that we can be certain none of the activations observed are due to differences in the content of the stimulus.
(4) Participants were not excluded based on handedness, although all subjects in the longitudinal study were right-handed.
(5) This section of the functional scan involved only auditory comprehension. Reading comprehension, which was added during the first year to the protocol, was given at the end of the scan.
(6) Each 30-s audio stimulus consists of unique utterances—no repetitions of content between or among languages.
(7) There is a 10 s rest period following each audio segment.
(8) A series of questions was administered immediately after the fMRI session, including the following list:
  ▪ Did you understand all of the utterances in the languages in which you are proficient?
  ▪ Did you understand any of the utterances in the 3rd and/or 4th languages? If so, approximate how often—less than 50%, more than 50%, *etc.*
  ▪ Is there anything that occurred during the imaging session that may have interfered with the listening comprehension process?
  ▪ All subjects answer a set of written questions for the auditory comprehension section and for the reading section. The experimental protocol is still in use and we do not want to bias future responses. Some samples, however, are given below.
    Do the students get help with reading? With speaking?
    Does the family like animals? What kind of apartment did they live in?
    What helps children deal with their emotions?
    What was the dog’s name?
(9) Finally, as part of the debriefing, the participants will be interviewed on the general content of what they heard and read and asked to provide information on their thoughts and sensations during the scanning process.

#### 2.2.3. Language Proficiency Testing

All LfMRI SLAM subjects have participated in multiple testing sessions using the official Russian language proficiency exam for the Russian Federation Ministry of Education (TRKI). TRKI is an instrument that meets the requirements of the language proficiency exams developed through the Council of Europe, CEFR ([2,3]). They have also been involved in course work and class examinations at both Duke University and St. Petersburg State University (Russia). The non-longitudinal subjects (MultiLing fMRI) also have been tested in Russian language using TRKI (an internationally-recognized evaluation tool used by the Russian Ministry of Education). During the study, five longitudinal subjects were tested at TRKI levels A2 and B1. Different types of proficiency (audition, reading, grammar/lexicon) were distinguished and measured and are included in the MANCOVA statistical model used to analyze these data and determine if proficiency plays a role in understanding the activations found in scans one through three. 

### 2.3. Subject Information

Subjects for LfMRI SLAM are five subjects (2 male and 3 females). The subjects began the study at 19 and 20 years of age. All subjects are right-handed.

The present paper covers scans conducted between April 2011 and April 2012. The project is ongoing, and a total of five scans per subject will be acquired by the end of the study in 2013. LfMRI SLAM subjects will be compared to a data set of 19 subjects scanned using the same protocol with varying degrees of proficiency in at least two languages after the study has ended. The common language for all subjects in LfMRI SLAM at the beginning is English, and Russian is the language being acquired during the study. None of the five subjects had any Russian language proficiency at the time of the first scan. 

Table 1 presents the stages of language acquisition the participants were enrolled in over the course of this section of the experiment. It also shows the relationship in time between the course of study, the scans, and the proficiency testing.

**Table 1:** Time table of fMRI scans and proficiency testing.

	2011					2012			
	Apr–Aug	Sep	Oct	Nov	Dec	Jan	Feb	Mar	Apr
**Course Work**		Intensive First Year				Intensive Second Year			
**Scan**	Scan 1				Scan 2			Scan 3	
**Test**				A2	B1 (a)	B1 (b)			

Formal Language Instruction (graded coursework conducted at Duke University and Saint Petersburg State University (SPSU):

Sept–Dec, 2011: Six contact hours per week for 13 weeks (at Duke); 20 contact hours per week for three weeks (at SPSU)
Jan–April, 2012: 16 weeks: six contact hours per week (at Duke)
May–June, 2012: Seven weeks, 120 contact hours total (at SPSU)

## 3. Analysis of fMRI Data

Preliminary analysis of the MultiLing 1 and LfMRI SLAM scans shows a much broader range of activation across both hemispheres in areas more broadly defined than the traditional Broca/Wernicke targets from the classical model. These results are in keeping with hypotheses and results found in [11,12,14]. Software for our fMRI image analysis includes FSL [72], SPM, FreeSurfer [73], and fScan [71,74,75].

Longitudinal experiments provide a unique window into individual as well as group differences and similarities in second language acquisition. Our data show a distinct difference in activation patterns between English, a language already mastered by the subjects, and Russian, a language being acquired by the subjects during the course of the study. When comparing 3 separate sets of scans across subjects in the conditions of English-rest and Russian-rest, we find that the number of activations across 55 ROIs in each hemisphere (following the Wake Forest University Pick Atlas) gives the following general pattern:

**English-rest:** Drop in number of regions of activation and mean level of activations across regions from first through third scan.
**Russian-rest:** Increase in number of regions of activation and mean level of activations from first through third scan; in some cases, individual regions of interest show a steady increase across scans, while in others, there is a slight drop between scans 2 and 3.

In order to begin our data analysis, we looked at the mean level of change in the same anatomical regions in the same conditions within a single subject over three separate scans (first in April, 2011, second in December, 2011 and third in April, 2012). One of the most significant points of comparison is between the English conditions, where no intensive language learning was occurring, and the Russian conditions, where there was intensive language acquisition. Also, the linking of proficiency and testing data for Russian language acquisition to the three scans for each subject is an important and essential step for contextualizing the fMRI results.

### 3.1. Regions of Interest

We include statistical data from comparative scan conditions in order to provide move quantifiable data from each scan. The regions of interest (ROI) that we focused on include the following (with left hemisphere Brodmann area *approximations*):

Left and right Medial Temporal Gyrus (MTG) BA 21
l/r Superior Temporal Gyrus (STG) BA 22
l/r Middle Frontal Gyrus (MFG) BA 46
l/r Inferior Frontal Gyrus (IFG) BA 44, 45, 47
l/r Postcentral Gyrus (PoG) BA 3, 1, 2
l/r Precentral Gyrus (PrG) BA four posteriorly, six anteriorly

(For more information on the Pick atlas used by Wake Forest University, see [76,77].)

These regions were selected based on the following principles: (1) regions that are frequently mentioned in the fMRI literature for sentence comprehension ([14] p. 68) or CSM literature ([9], pp. 107–111) and (2) regions that showed significant change across the language acquisition scans.

### 3.2. Longitudinal Comparisons across English and Russian Conditions across Subjects

**Figure 1:**
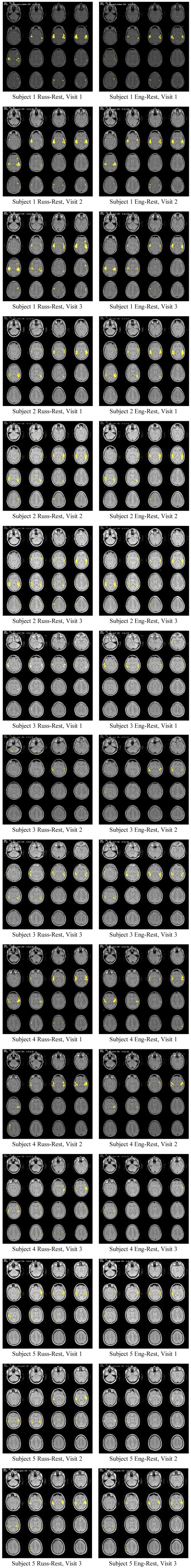
Functional activation t-maps (thresholded at *t* > 4), superimposed onto proton-density anatomical images, subjects 1 through 5, for three visits in two conditions (Russ-Rest, Eng-Rest).

**Figure 2:**
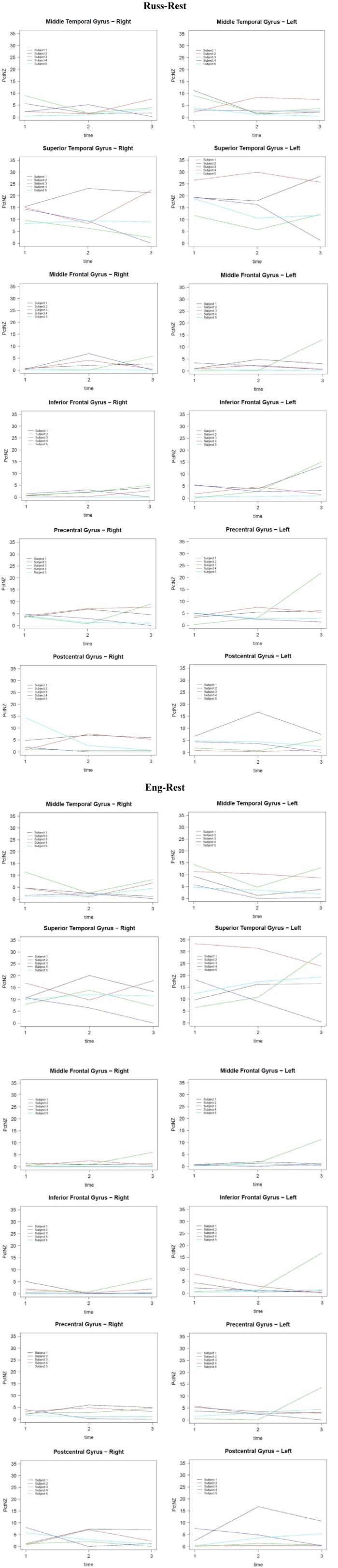
Percentage of nonzero voxels (PctNZ) for five subjects by ROI and hemisphere over three scans (time), for conditions (Russ-Rest, Eng-Rest).

Data across the five longitudinal subjects demonstrates a significant difference in the results obtained from English conditions in comparison with those obtained from Russian conditions. Figure 1 shows functional activation t-maps (thresholded at *t* > 4), superimposed on the subjects’ own overlays of functional images onto proton-density anatomical images. Figure 2 shows plots of the percentage of non-zero voxels per region for English-rest and Russian-rest for ROIs in Subjects 1–5 of the longitudinal study.

### 3.3. MANCOVA Analysis

The multivariate analysis of covariance is based on proficiency testing measurements and fMRI ROI measurements (12 regions) generated under two pairs of conditions: (1) English-rest and (2) Russian-rest. The raw data are the percentage of voxels in each region whose activity levels are above threshold. Additionally, to provide an internal benchmark, we also used fMRI readings from the Middle Occipital Gyrus, a region which would not be relevant in a listening comprehension condition.

The primary model used in this analysis was MANCOVA, where the vector of responses (the percents of non-zero voxels by time, region, hemisphere, and subject) is modeled as the additive effects of two categorical variables (hemisphere and region) and one continuous covariate (proficiency score):

**Y** = mean + region effect + hemisphere effect + score effect

An earlier exploration allowed a two-way interaction between hemisphere and region, but this was not significant. Also polynomial effects in score were not found to be significant. The MANCOVA model is appropriate since this experiment entails repeated measures on the same set of individuals, so that performance over time is likely to be correlated (Morrison 1990 [78]).

The analysis treated the two pairs of conditions (*i.e.*, English-rest, Russian-rest) separately. Although all four conditions could be handled simultaneously in one MANCOVA model with more complicated repeated measures structure, we analyzed the two conditions by fitting the same form of the MANCOVA model each time. This simplifies the interpretation of the results, and reduces the risk of misleading results due to failure of model assumptions (such as non-normal residuals) or some aberrant situation in one of the test conditions.

In reporting the results from these two MANCOVA models, we use Pillai’s trace to test all hypotheses. Alternative statistics, including Wilks’ lambda, Hotelling’s trace and Roy’s largest root, found nearly identical results. We also note that tests of sphericity or specific pattern matrices for the correlation structure were generally rejected, implying that the covariance matrix is complex.

#### 3.3.1. Primary Results of the Analysis

The primary results of the analysis include the following points:

1. The score effect for the repeated measure is significant for the conditions Russian-rest. *Pillai’s trace* has *p*-values where *p* = 0.01. 

As expected, the score effect is not significant for English-rest, where the *p*-value equals 0.47. This supports the research hypothesis that language acquisition is associated with characteristic activations found in the Russian conditions. Furthermore, the fact that it was insignificant for the English-rest condition strongly supports the belief that non-normal residuals are not distorting the analysis in any important way.

2. The time effect is significant; activation levels change across the three different sets of measurement.

3. For the *between-subjects effects* (region, hemisphere, and score), all effects were significant in the English-rest and Russian-rest conditions, where the largest *p*-value among the six was 0.04. The interpretation of these results is that both region and hemisphere effects appear in the fMRI data (although their interaction term is not significant). 

The score effect is significant for both the ***within effect*** (an interaction between score and time) and the ***between effect***. The *within effect* shows that score effect changes over time in the Russian-rest condition, but **not** in the English-rest condition as is predicted by the research hypotheses of this study. The *between effect* means that the average amount of fMRI signal varies with score, where larger scores imply more activation in five ROIs and less activation in one ROI.

#### 3.3.2. Secondary Results of the Analysis

Secondary results of the analysis include the following results:

1. The time effect is significant for Russian-rest; average activation levels change across the different sets of measurements.
2. The time effect is not significant for English-rest; average activation levels do not change across the different sets.
3. There is a significant hemisphere effect.
4. The Middle Occipital Gyrus, used as an internal statistical standard, shows a lack of effect as expected.
5. Different regions show variation in activation patterns.

In interpreting these results, we note that the covariate measurement used for the proficiency score was the average of the first set of three separate test scores (auditory comprehension, reading, and grammar at the CEFR B1-level). A more complex MANCOVA model could also use the proficiency testing scores acquired before the second fMRI session, provided that a sensible imputation of the non-existent scores at the first fMRI session could be obtained (assigning a zero to everyone before the Russian language training begins would almost certainly ensure a spuriously exaggerated score effect).

## 4. Towards an Explanation of Bilaterality of Language

One of the central points that has been criticized consistently by the contemporary neurolinguistic community, including those researchers who use fMRI as one of their main imaging techniques for studying language and brain, is the lack of attention that has been paid to the right hemisphere (especially [11,15,16,79]). Our data consistently shows important activations involving both hemispheres with some interesting differences across subjects. Future data analysis will include additional statistical modeling to attempt to provide an explanatory basis for understanding more clearly the role of both hemispheres in language acquisition and maintenance across levels of proficiency.

## 5. Discussion

There are many debates about what fMRI studies of language can and cannot show. Some of the more critical voices can be found in the works of de Bot (2008) and Paradis (2004) [1,14]. The present study provides data that support the use of fMRI in understanding language acquisition and maintenance and demonstrates that proficiency is a statistically significant effect. We also believe that the evidence supports the development of more fMRI **longitudinal **studies for the study of bi- and multilingualism and second language acquisition where proficiency data are included as a key component of the analysis. The drawbacks to fMRI longitudinal studies include high cost and potential subject attrition. The importance of multivariate analysis models including covariance become essential in studies where proficiency measurements and other behavioral empirical data are acquired in conjunction with scanning data. There is no doubt that the presence of these additional data points strengthens the interpretations and analysis of results obtained using fMRI.

Another aspect of our study that is different from previous studies is the inclusion of specific testing and proficiency data that (1) are the same for all of the subjects in the study and (2) based on proficiency testing that is regularized across the languages of Europe and recognized by the Council of Europe and articulated by the Common European Framework of Reference and targets grammar/lexicon, reading, listening comprehension, speaking and writing. The inclusion of this data enhances our understanding of the data acquired using fMRI technology. 

One of the concerns raised by critics of fMRI studies (and PET) is the difficulty in comparing the results across studies (especially [80,81]). While we recognize this point, our conclusions are in line with data collected from a large range of fMRI and CSM language studies. They support a broader understanding of bilateral activations for language(s) and suggest a series of regions of interest that may be considered in future studies. Development of more longitudinal fMRI studies studying bi- and multilinguals and second language acquisition at the discourse level will provide a strong basis for deepening the relevance of imaging experiments that focus on language and languages. 

While it is not necessarily the case that CSM data should or do correspond to fMRI acquired data in general, we believe that both techniques provide important information about neurological representations of human language. By using regions of interest that are identified across these techniques, it may be possible to reach a new level of understanding of the relationship between localization and language function in the brain.

The fact that the LfMRI SLAM data show both variability and invariability across and within subjects can be understood from several perspectives. While the participants in the longitudinal study had very similar acquisition of a second/third language, Russian, there are still important individual differences in motivation, aptitudes, attitudes toward Russian language and culture, other languages learned, and how one intends to use the language in the future. Here again, the linguistic community has been very critical of the “merging” of subject data in order to make strong localization-based claims that ignore intra-subject variation. We have attempted to provide more information at the individual subject level across time and be careful not to explain away differences between subjects.

Finally, the importance of bilateral language areas has been known for many years, but still remains an understudied phenomenon (*cf.* [11]). Our data indicate that there is merit in pursuing more in-depth study of bilateral activations in future studies. We will also revisit this issue in our continued analysis of reading and auditory comprehension as our longitudinal study progresses.

## 6. Conclusions and Future Directions

The present paper is an introduction to a robust set of data acquired longitudinally using both fMRI and behavioral and proficiency data on a set of five subjects who begin their intensive study of Russian during the study. Coordination of proficiency testing and fMRI scanning of subjects allow for a unique opportunity to analyze to what degree fMRI may track language acquisition within subjects longitudinally. The behavioral and proficiency data make it possible to provide empirically valid information about the achievements of the subjects in a range of measurements that are available by task (listening comprehension, reading, grammar/lexicon) as a component of the analysis of the fMRI scan data for a listening comprehension task. 

Using a multivariate analysis of covariance based on proficiency testing measurements and fMRI ROI measurements (12 regions) under the conditions of English-rest and Russian-rest, a score effect for the repeated measure yields a significant *p*-value, where *p* = 0.01. This result supports the fundamental research hypothesis that language acquisition is associated with characteristic activations found in the Russian conditions. Furthermore, the fact that the p-value is insignificant for the English-rest condition (where *p* = 0.47) strongly supports the belief that non-normal residuals are not distorting the analysis in any important way. Finally, time effect is significant for the Russian conditions and shows activation levels changing across the three sets of longitudinal measurements.

The importance of understanding *invariance in variation* has been one of the central concerns of theoretical linguistics of the 20th and 21st centuries. The construction and conducting of imaging studies that include protocols of language that are not only ecologically valid and coupled with behavioral and proficiency data, but also allow for multiple comparisons across and within subjects longitudinally, may provide a new perspective on how to answer some of the most challenging issues about brain and language, as well as formulate new questions that can deepen the research paradigms in cognitive and neurolinguistics. 

In addition to the findings emerging from our initial analysis of the fMRI SLAM project discussed above, there are several important points to be pursued in subsequent papers using these data. Specifically, our project is still in progress and an additional two scans are scheduled for acquisition over 2013, which will result in a total of five scans per subject. We also will work to further contextualize our findings with the general fMRI literature on first and second language and multilingualism, as well as consider other approaches, including CSM, to provide a broader basis for analysis and contextualization of fMRI results of languages. The inclusion of more studies of “comprehension of complex sentences” ([82] p. 309) or, in our case, ecologically-valid discourse, may provide more robust results than studies that focus on single-word stimuli.

While the present paper is only an introduction to longitudinal study of multilingualism and second language acquisition, it serves as a first step in attempting to construct a prescient model that more deeply integrates behavioral information, empirical testing and proficiency data with computational data provided from fMRI studies embedded in appropriate statistical analysis.
